# Electromyography-Based Control of Lower Limb Prostheses: A Systematic Review

**DOI:** 10.1109/tmrb.2023.3282325

**Published:** 2023-06-07

**Authors:** Bahareh Ahkami, Kirstin Ahmed, Alexander Thesleff, Levi Hargrove, Max Ortiz-Catalan

**Affiliations:** Center for Bionics and Pain Research, 43130 Mölndal, Sweden, and also with the Department of Electrical Engineering, Chalmers University of Technology, 41296 Gothenburg, Sweden; Center for Bionics and Pain Research, 43130 Mölndal, Sweden, and also with the Department of Electrical Engineering, Chalmers University of Technology, 41296 Gothenburg, Sweden; Center for Bionics and Pain Research, 43130 Mölndal, Sweden, also with the Department of Electrical Engineering, Chalmers University of Technology, 41296 Gothenburg, Sweden, and also with Integrum AB, 43153 Molndal, Sweden; Department of Physical Medicine and Rehabilitation, Northwestern University, Chicago, IL 60611 USA, and also with the Regenstein Foundation Center for Bionic Medicine, Shirley Ryan AbilityLab, Chicago, IL 60611 USA; Center for Bionics and Pain Research, 43130 Mölndal, Sweden, also with the Department of Electrical Engineering, Chalmers University of Technology, 41296 Gothenburg, Sweden, also with the Operational Area 3, Sahlgrenska University Hospital, 41345 Gothenburg, Sweden, and also with Bionics Institute, Melbourne, VIC 3002, Australia

**Keywords:** Electromyography (EMG), pattern recognition, lower limb amputation, control architecture, control algorithms, movement intention recognition

## Abstract

Most amputations occur in lower limbs and despite improvements in prosthetic technology, no commercially available prosthetic leg uses electromyography (EMG) information as an input for control. Efforts to integrate EMG signals as part of the control strategy have increased in the last decade. In this systematic review, we summarize the research in the field of lower limb prosthetic control using EMG. Four different online databases were searched until June 2022: Web of Science, Scopus, PubMed, and Science Direct. We included articles that reported systems for controlling a prosthetic leg (with an ankle and/or knee actuator) by decoding gait intent using EMG signals alone or in combination with other sensors. A total of 1,331 papers were initially assessed and 121 were finally included in this systematic review. The literature showed that despite the burgeoning interest in research, controlling a leg prosthesis using EMG signals remains challenging. Specifically, regarding EMG signal quality and stability, electrode placement, prosthetic hardware, and control algorithms, all of which need to be more robust for everyday use. In the studies that were investigated, large variations were found between the control methodologies, type of research participant, recording protocols, assessments, and prosthetic hardware.

## I. Introduction (Background)

IN THE United States, it is estimated that by 2050 there will be 3.6 million people living with amputation compared with 2.2 million people today (2021) [1]. In developed countries, disease accounts for most amputations and the majority occur in the lower limb (LL) [1], predominantly due to the prevalence of diabetes mellitus and associated vascular complications [2]. By the turn of this century, 93% of all lower-limb amputations were the result of vascular disease (28% transtibial, 26% transfemoral [3]). In many low and middle income countries, trauma has been documented as the primary cause of limb amputation [4], [5], [6].

In 2017, it was estimated that limb amputation due to trauma is 57.7 million. of people with traumatic amputation, an estimated 31.7% had unilateral lower limb amputations (28.9 million, Uncertainty Interval (UI) =26.9–32.1), 19.6% had unilateral upper limb amputations (11.3 million, UI = 10.6–12.1), 19.1% had bilateral upper limb (11.0 million, UI = 10.3–11.9), and 11.1% had bilateral lower limb (6.4 million, UI = 5.9–7.0) [4].

Unlike upper limb prostheses, no commercially available lower limb prosthesis integrates signals from the user’s neuromuscular system for control. The high number of lower limb amputations, combined with the lack of neuromuscular-controlled lower limb prostheses, highlights an area of opportunity to develop more intuitive, reliable, and functional bionic legs. There are currently three categories of lower limb prostheses: passive, semi-active, and active. Passive prostheses are entirely mechanical, while semi-active prostheses use microprocessor systems that measure information from mechanical (non-bioelectric) sensors to modulate artificial joint impedance usually via hydraulics or pneumatics. Finally, active prostheses provide propulsion or “power” using actuators (*i.e.*, motors at the joints) to compensate for lost musculature [7] and are also controlled using a microprocessor. Harnessing neuromuscular information to control semi-active and active prostheses can provide a more biomimetic, functional, and superior experience for the users. EMG signals can be recorded non-invasively using electrodes placed on the surface of the skin (sEMG) or invasively using surgically implanted electrodes (iEMG) [8]. Unless the amputation was accompanied by nerve injury or other motor impairments, motor commands to residual muscles, previously responsible for actuating the missing joint(s), can still be generated voluntarily by the patient. Neuromuscular controlled prostheses can take advantage of this by recording EMG signals from the user’s residual muscles to control powered artificial joints [9]. However, with proximal amputations there may be too few muscles remaining to intuitively control the missing joints. Surgical techniques such as Targeted Muscle Reinnervation (TMR) [10] or Agonist-antagonist Myoneural Interface (AMI) [11] have proven successful in overcoming this problem by creating new EMG sources for prosthetic leg control. Moreover, a variety of signal processing and machine learning algorithms have been employed to further improve the neural decoding of motor commands in individuals with lower limb amputations [12], [13], [14], [15], [16], [17].

A limitation in the use of sEMG for prosthetic control is the long-term stability of the signals. Conversely, iEMG produces signals with long-term stability and has shown to be a clinically viable solution in individuals with upper limb amputations [18], [19], [20], [21]. The lack of neuromuscular integration in lower limb prosthetic development to date can be attributed to several factors. Firstly, the consequence of unreliable control in a lower limb prosthesis is serious; error may result in a fall and subsequent injury. Secondly, non-invasive recordings of sEMG using suspension sockets are challenging due to pistoning (vertical movement inside the socket) and changes in pressure between the prosthetic socket and residual limb. Thirdly, active devices, which have the capability to perform more activities, such as repositioning the joints to prepare for transfers, have only recently become commercially available. These are the categories of devices that arguably have the most to gain from a neuromuscular control paradigm.

Direct skeletal fixation of an implant harnessing the biological process of osseointegration, as opposed to suspension by a socket [22], can provide a gateway for a permanent wired connection between implanted electrodes and the prosthesis [18], [23]. In participants with direct skeletal fixation, problems caused by pistoning and changes in pressure between the socket and residual limb are alleviated, thus sEMG can provide reasonable control.

In this systematic review, we provide an overview of pioneering and state-of-the-art research in the field of lower limb prosthetic control using EMG signals. Two reviews including EMG control were published during the preparation of this article and have been included herein for completeness [24], [25]. Fleming et al. [24] conducted a topical review, and Cimolato et al. [25] presented a systematic review including 56 articles. Our systematic review covered a larger volume of articles (121) and provides further division of control strategies using EMG. We investigated and categorized control methods into three main groups: 1) Direct control, where the modulated EMG activation directly and continuously relates to the ankle or knee joint actuator either in a virtual environment or to control a prosthesis; 2) Model-based control, in which body sections are modeled as rigid segments connected by rotational joints and driven by joint actuators (which model muscles) [26]; and 3) machine learning control, where a decoder is trained to distinguish EMG patterns between different locomotion modes, gait phases, or leg movements. This method of categorization differs from the review by Fleming et al. [24] highlighting model-based control in its own category to reflect its increasing popularity. Challenges in controlling lower limb prostheses with EMG are also summarized and discussed.

## II. Method

The systematic review followed the Preferred Reporting Items for Systematic Reviews and Meta-Analyses (PRISMA) [27].

### A. Search Strategy and Eligibility Criteria

Four online databases (Web of Science, Scopus, PubMed, Science Direct) were searched until June 2022 for peer-reviewed, English-language research articles, and conference proceedings published at any time. The search was conducted using the keywords: *prosthetic* OR *prostheses* OR *prosthesis* OR “*artificial limb*” AND ((“*lower limb*”) OR *leg* OR *ankle* OR *knee*) AND *control* AND (Electromyography OR *EMG* OR *neural*).

To be included, the studies must have recorded EMG signals, and used the recorded signals to control an ankle, leg, virtual object, or to classify gait intent or locomotion modes. We excluded publications that were not in English, were patents, books, or abstracts. Similarly, articles describing control methods for an exoskeleton, or EMG for general rehabilitation purposes, were excluded. Furthermore, publications that focused exclusively on surgical methods, developments or improvements of leg prostheses, control hardware, or firmware, were excluded. Studies on non-human subjects and studies using biosensors other than EMG (such as EEG) were also excluded.

### B. Selection Process

**Fig. 1.**
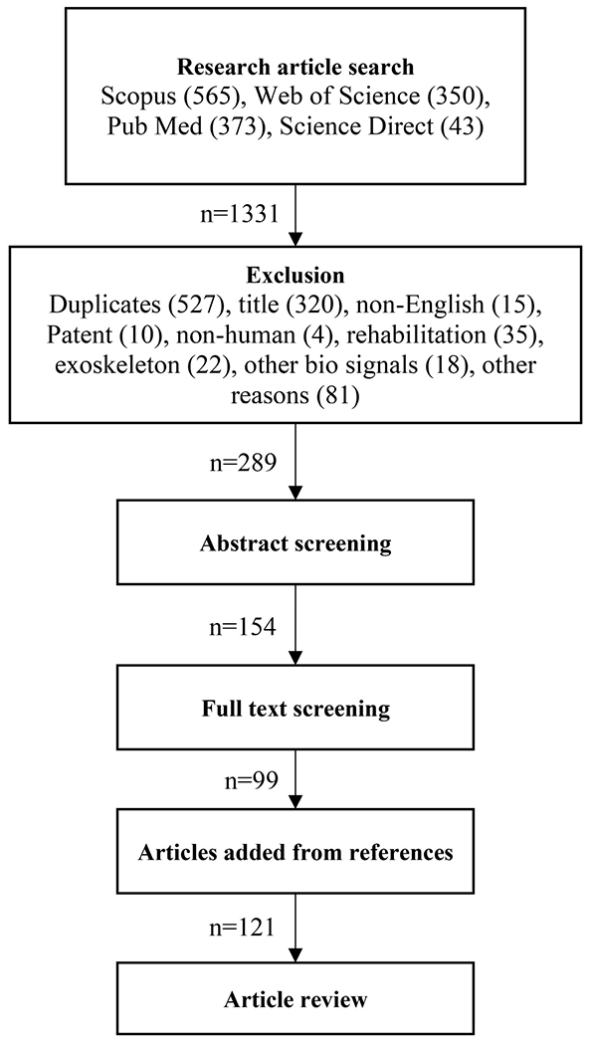
Flowchart of the systematic literature review. Searching keywords in four databases resulted in 1331 papers. After exclusion based on exclusion criteria, abstract and full text screening, 121 papers remained.

The literature search and article screening procedure were performed according to the sequence of steps shown in Fig. 1. A total of 121 articles passed the screening procedure and were thus assessed in detail and included in the review.

## III. Results

### A. Research Participants

**Fig. 2.**
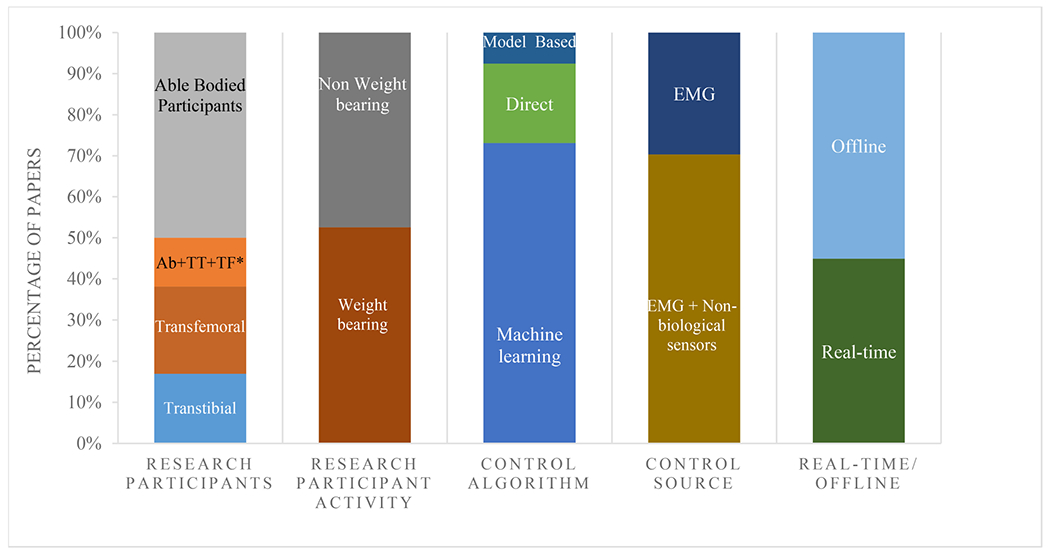
Proportion of studies in different categories. Studies are divided into five categories: research participants, research participant activity, control algorithm, control source, and real-time or Offline processing of data. TF: Research participants with transfemoral amputation, TT: Research participants with transtibial amputation, Ab: Able-bodied research participants.

Of the 121 included articles, 59 articles (~50%) included able-bodied research participants, 50 articles (~40%) included individuals with amputation, and 12 articles (~10%) included research participants from both cohorts (Fig. 2).

### B. Research Participant Activities: Assessments

**Fig. 3.**
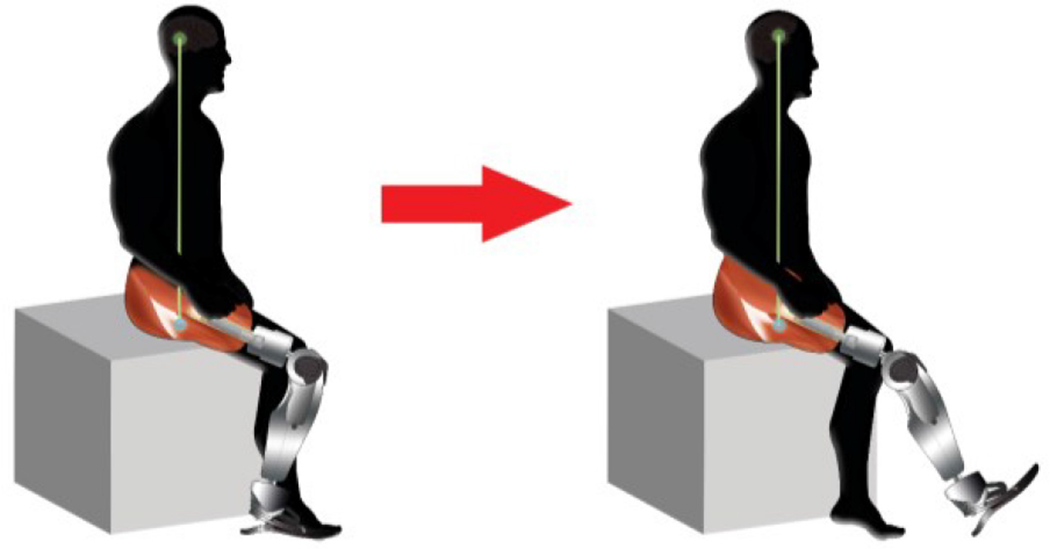
Seated knee extension and flexion as an example of a non-weight bearing activity.

**Fig. 4.**
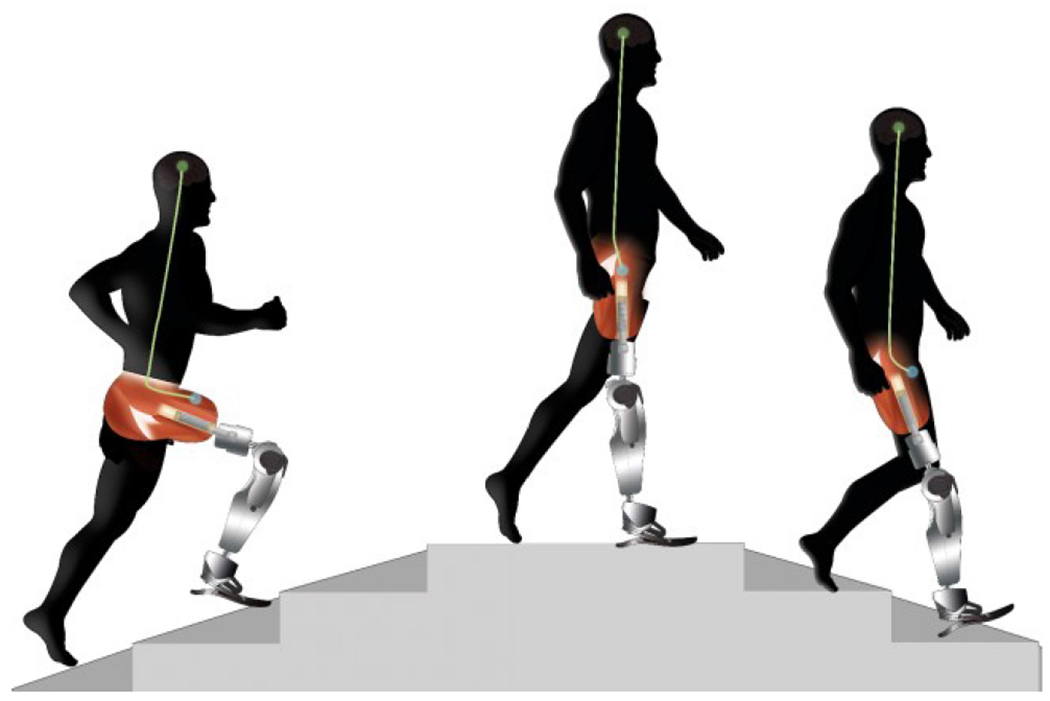
A transition from stair ascent to walking and from walking to stair descent as an example of a weight bearing movement to classify locomotion modes.

Activities were divided into those with research participants performing non-weight-bearing (Fig. 3) and weight-bearing activities (Fig. 4). In more than 50% of the included studies, research participants were asked to perform weight-bearing activities. Of the weight-bearing activities, gait studies were the most common in which the goal was to classify the locomotion mode (*e.g.*, walking, stair ascent, etc.) or functional phase of gait [28], [29], [30]. The stance (support) phase of gait comprises 60% of the gait cycle and are split into: heel contact, loading response, mid stance, terminal stance, and push off. The swing phase of gait comprise 40% of the gait cycle and are made up of initial swing, mid swing and terminal swing [31].

The non-weight bearing activities (Fig. 3) in the reviewed studies were of variable format and the research participants were asked to perform movements with their phantom (individuals with amputation) or intact (able bodied research participants) limb. In some studies the research participants were asked to mimic pre-programmed motion trajectories [32], [33], [34], [35], [36] or to perform isolated single joint movements (one degree of freedom (DOF)) [37], [38], [39]. In other studies the participants controlled a virtual object in a 2D or 3D space [11], [40], [41], [42], [43].

### C. Myoelectric Sources

The muscles used for EMG acquisition differed between the studies depending on research participant anatomy, type of study, and the joints to control. Frequently reported muscle groups used for EMG acquisition in research participants with transfemoral amputation were: semitendinosus, biceps femoris, tensor fasciae latae, rectus femoris, vastus lateralis, vastus medialis, sartorius, adductor magnus, and gracilis [10], [28], [39], [44], [45], [46], [47], [48], [49], [50], [51], [52], [53], [54], [55]. In research participants with transtibial amputation, we found the gastrocnemius medial and lateralis, tibialis anterior muscle to be the most frequently reported muscles used for EMG acquisition [32], [33], [43], [56], [57], [58]. The number of electrodes varied but it was as high as 192.

### D. Non-Biological Sensors

**Fig. 5.**
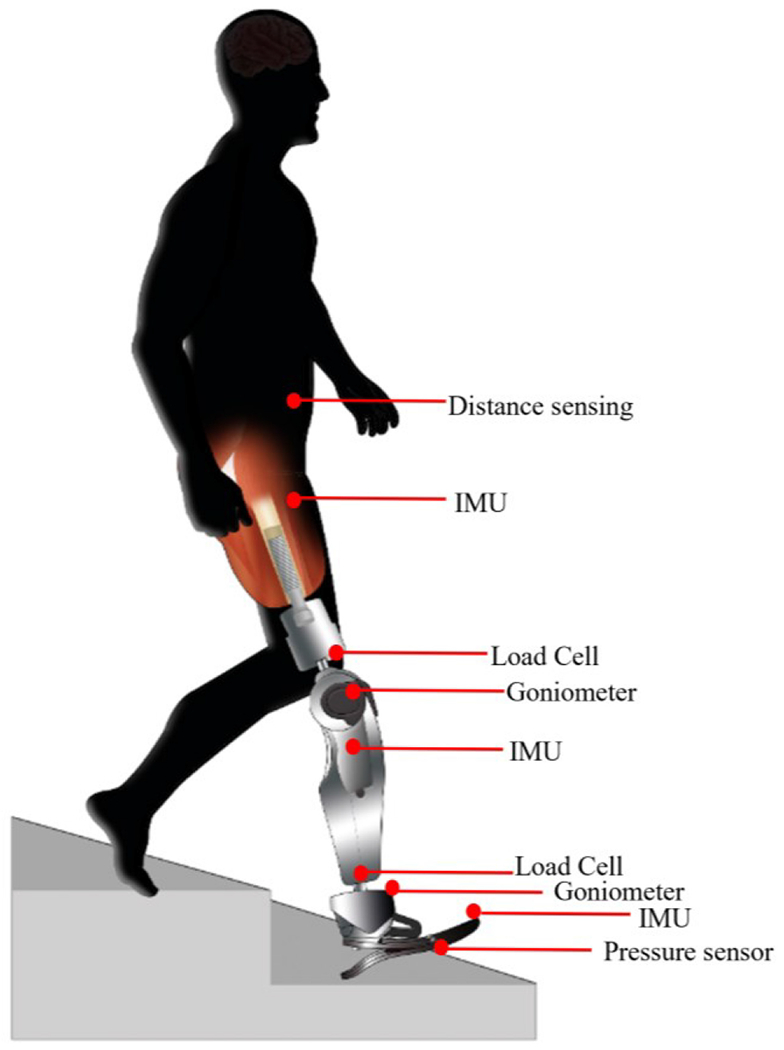
Illustration of different non-biological sensors and their placements. Sensors reported in the studies were distance sensors, IMUs, loadcells, goniometers, and pressure sensors. They were used either in combination or alone.

To date, sensors providing mechanical information have been the only source of information for lower limb control algorithms in commercial devices. Inertial Measurement Unit (IMU) sensors provide useful control feedback for lower limb support or swing gait stage detection and are used alone or in combination with depth sensors and goniometers [59], laser distance sensors [34], or loadcells [60]. A foot switch sensor can indicate the temporal gait stage determined by ground contact and was a commonly reported sensor in the literature [49], [58], [61], [62], [63], [64], [65], [66]. An overview of sensors and common placements can be found in Fig. 5 sensors can be placed on the amputated side or on both legs [53] but the majority of studies reviewed used sensors only on the amputated side. This makes sense from the bionic leg control perspective when solely harvesting information from mechanical sensors, however from a person-centered approach, a control method that additionally receives input from biological signals is logical. EMG signals have been used in research devices in combination with mechanical sensors to inform the gait phase or locomotion mode. Performance of pattern recognition algorithms was found to improve when EMG was added [47], [51], [52], [59], [67], [68].

### E. EMG Control Methods

The use of EMG in the control of lower limb prostheses has become a fast-growing research area. Here we further divided in direct control, model-based control, and machine learning control. The next section provides an overview of each of these methods.

#### 1) Direct Control:

This method uses a modulated EMG signal to activate the ankle or knee joint actuator directly and continuously either in a virtual or prosthetic device [69]. Direct control studies quantify performance using outcome metrics such as ankle/knee prosthetic joint angle or distance moved between target and a virtual object. While direct control was predominantly reported in the reviewed studies involving transtibial amputees, it has also been utilized in studies with participants with transfemoral amputation. In these cases, EMG signals were recorded from proximal muscles to determine knee torque during the stance phase [70].

Nineteen out of 121 studies used the method of direct control of which 13 controlled an ankle joint actuator and 6 controlled a knee joint actuator. A full list of the reviewed studies using direct control are shown in Table I.

The activation of the prosthesis can be designed to be proportional to the magnitude of the EMG signal, this is proportional control. A frequently reported experiment was to move a virtual object in a 2-D space directly and proportionally to the EMG signal while performing plantar and dorsiflexion [11], [33], [40], [41], [71]. Fleming et al. designed a virtual pendulum proportionally controlled by EMG signals and showed an anticipatory postural adjustment in participants with transtibial amputations [43]. The computational cost is relatively low in direct control, even including proportionality, and therefore it is a common and valuable method of control for devices with lower degrees of freedom. However, obtaining enough independent signals to directly control several degrees of freedom is challenging, where model-based and machine learning approaches are commonly used (Table I).

#### 2) Model-Based Control:

Model-based control is the second category of EMG control frequently reported in the reviewed literature. Here body segments are modeled as rigid bodies connected by rotational joints and driven by joint actuators (which model muscles) [26]. Hoover and Fite modeled the lower limb muscles as parallel spring-damper systems, where co-activating the muscles modulated the net mechanical impedance of the joint [72]. Furthermore, they produced a knee model in which the knee moment was a function of the thigh EMG [73]. In a different approach, Hargrove et al., designed an active-reactive model where joint torque was determined by the difference in agonist/antagonistic muscle pair activations, and joint impedance was determined by the sum of the agonist/antagonist muscle pair activations [46]. Minor et al., used an autoregressive model with a low frequency EMG envelope as another way to predict the knee moment [74]. Most modeling methods relied on a motion capture system within a specialist gait lab. Whereas specialized equipment restricts the exploitation of the model-based control method, it does provide a well validated basis for model creation. Additionally, it allows for generalizable models that can be fitted to new subjects unlike personalized machine learning methods (see Section III–E3). For example Cimolato et al. developed a body model from sEMG signal and IMUs to control a prosthetic leg during gait [75]; these results were comparable to those obtained using motion capture systems.

#### 3) Machine Learning Control Methods:

##### a) Overview:

Machine Learning methods can be used for detecting different gait phases, locomotion modes, and transitions between locomotion modes. They also can be used for decoding non weight bearing movements such as knee or ankle flexion/extension [76]. Control methods based on machine learning do not need an *a priori* model but often require data to train a classifier or decoder. In brief, the decoding of motor volition using EMG signals can be divided into 1) pre-processing, 2) feature extraction, 3) classification, and 4) post-processing.

**Fig. 6.**
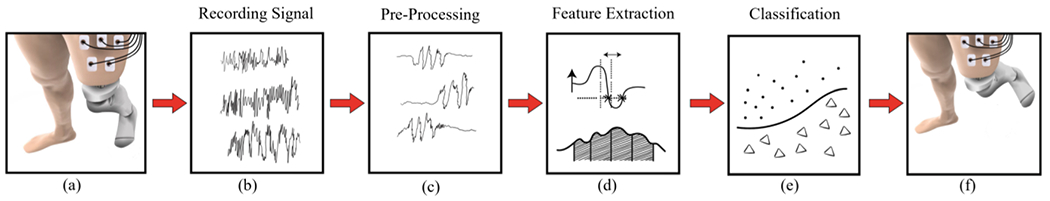
Data processing in a machine learning EMG controlled prosthetic leg. a) Surface electrodes on the residual limb of a transfemoral osseointegrated participant, b) EMG signal obtained from the residual limb, c) Filtering and segmentation of the EMG signal d) Feature extraction from the EMG signal in time domain and frequency domain, e) Classification of movement intention from the extracted features, f) The prosthesis moving according to the intention of the participant.

In the pre-processing phase, the raw EMG signal undergoes *filtering* and *segmentation* in “time windows” to remove noise from the raw data and to parse relevant portions of the continuous EMG signal (Fig. 6c), respectively. The continuous EMG can be segmented in overlapping and non-overlapping time windows. In the overlapping method, two consecutive windows have an overlap time that is less than the length of the time windows themselves. The time between the beginning of a time window and the next is known as the increment step. Finding the optimal length for a time window and the increment step to the next is important because long windows and steps reduce the real-time responsiveness of the system, while short windows may lack information for an accurate prediction. As a result, a balance between using more information for decoding and responsiveness must be maintained. Segmentation is followed by a feature extraction process where characteristics of each time window are extracted to form a set of features or feature vector (Fig. 6d). EMG features can be extracted in either time, frequency, or time frequency domains. An example of a time-frequency extraction method is the wavelet packet transform [74], [75]. After feature extraction, dimensionality reduction can decrease the number of features processed by classifiers to supply only the most relevant information.

In the classification phase, the feature vector is transferred to a pre-trained decoder (*e.g.*, Support Vector Machine) to classify intended movement (Fig. 6e). One method used often in upper limb control is continuous classification [79], [80]. Using this method, classification takes place regardless of the state of the arm, hands, and fingers. In lower limb control, the EMG signal in gait is “quasi-cyclic” since gait phases from heel strike to toe off are approximately cyclical. Consequently, it is sensible to perform locomotion classification at specific events instead of continuously and therefore Phase-Dependent Classification is commonly employed in lower limb control.

The post-processing phase is where the effect of misclassifications can be attenuated using filtering techniques (*e.g.*, majority voting or using a velocity ramp [81]).

#### b) Results of the reviewed publications: (Pre-Processing methods):

##### Filtering:

Of the 121 publications using EMG, 81 applied a 20 – 500 Hz bandpass filter to remove unwanted low frequency artifacts and high frequency noise [82]. Filtering should be done in a way to avoid the loss of useful data or cause any unwanted changes such as obscuring an adverse event. This is important when trying to avoid stumble and falls.

##### Segmentation:

Most of the studies used a window length of 150 - 300 ms with an overlap of 20 - 50 ms [83], [84], [85]. In almost all the included studies in the machine learning category, the window length was constant throughout the experiment. There were a few exceptions to this, in which varying window lengths were employed, for example, Miller et al. introduced a segmentation method with three EMG sub-windows per gait cycle (one gait cycle was defined as ipsilateral heel strike to ipsilateral push off): 1) heel strike to heel strike + 200 ms, 2) push off − 300 ms to push off, and 3) push off to push off + 100 ms [58].

##### Feature Extraction:

In the reviewed publications, the most often reported features were the average absolute value, zero crossings, number of slope sign changes, and waveform length, which are all time domain features [49], [54], [86], [87]. Use of frequency domain features were reported less frequently, this may be related to their computational cost. Among the studies that reported dimensionality reduction, principle component analysis (PCA) was often the method used [35], [88] where a set of variables was transformed to a lower dimension set of uncorrelated variables (principal components). Farrell and Herr used a distinct dimension reduction method called the wrapper method [64]. This is a sequential-forward-search that finds a subset of features that maximizes the leave-one-out cross-validation performance on the training.

#### Classification:

##### Phase-dependent classification:

**Fig. 7.**
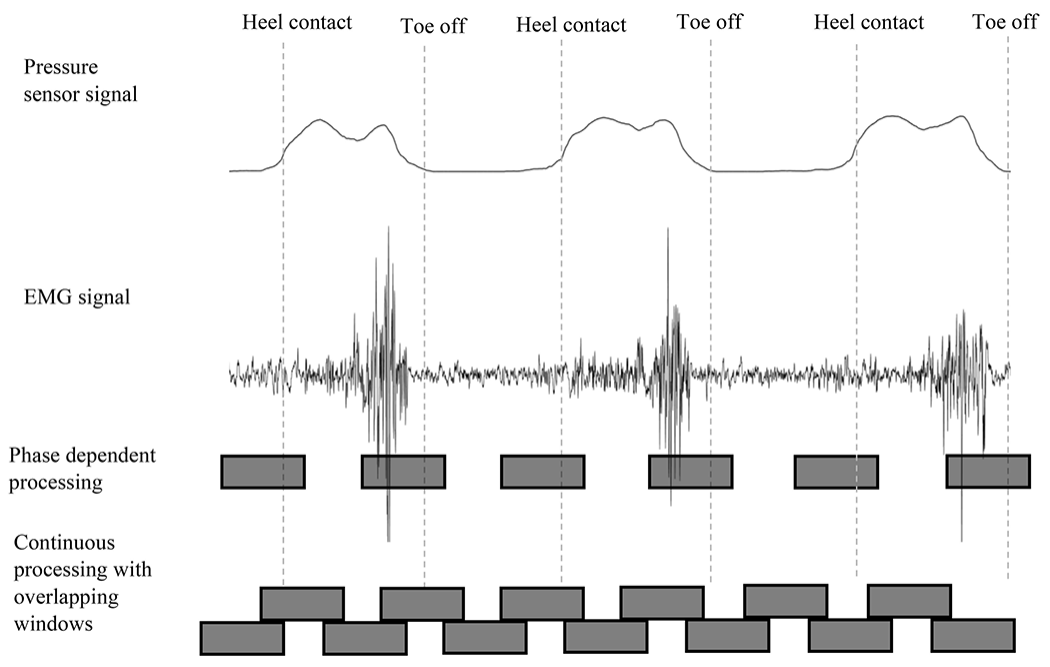
Illustration showing the difference between phase-dependent and continuous methods of classification. In the phase-dependent classification, a time window at every pre-defined gait phase (toe off, heel contact) is classified, while in continuous classification, every consecutive window is classified.

Phase dependent classification (Fig. 7) classifies the signal features at specific moments during every previously defined phase (*e.g.*, heel strike or toe off). A signal window around the defined phase is classified. All reviewed publications employing this method use a similar approach, with slight differences in the number of gait phases, windowing, or type of classifier. For example, Young and Hargrove. and Spanias et al. used eight different phases of the gait cycle; 0%, 25%, 50%, and 75% of swing and 0%, 25%, 50%, and 75% of stance [29], [45]. Zhang et al. used four clinically defined gait phases: heel contact, mid stance, terminal stance and swing [88]. Huang et al. used toe off and heel contact [49]. Classification can be performed either before or after each of the selected gait phases. Furthermore, as shown in [49], [58], [59], [87], [89] it is possible to have two distinct classifiers, for both a window before and a window after a gait phase. Of the publications reviewed, Linear Discriminate Analysis (LDA), Support Vector Machine (SVM), a combination of LDA and Bayesian methods, and neural networks have been used to predict the next locomotion mode (see Table I). A limitation of phase-dependent classification is the requirement for a gait phase input (windowing is relative to the phase changes). Gait phase can be obtained using non biological sensors (such as a load cell), discussed separately in this review. A complete list of publications reviewed using phase dependent classification can be found in Table II.

##### Continuous classification:

Despite being a less frequently reported method of classification in lower limb control, several studies we reviewed did report it in non-weight bearing movement (presumably since they are non-cyclic) [35], [40]. Unlike phase dependent classification, continuous classification is possible without the input of gait phase state (Fig. 7, bottom row). The gait phase can be recognized [66], [90], instead of predicting the locomotion mode or user motion intent [91]. This offers the distinct advantage of not relying on sensor interpretation of gait phase, but they cannot be used to detect different locomotion modes. From the EMG signal, windows are selected and processed with a fixed pattern. Gaps or overlaps can be considered between windows. A complete list of publications that used continuous classification can be found in Table III.

##### Post Processing:

Majority vote was a frequently reported method for reducing the effect of misclassification in the literature we reviewed. For instance, if a five-point majority vote was used, transition did not occur until at least three of the previous five overlapping windows agreed on the new locomotion mode. Other studies employed a voting scheme which increased the number of voting decisions each time a rare transition was identified, such as the transition from stair ascent to stepping over an obstacle [102], [103]. In these studies, the regular number of voting points was five; the voting length increased to 15 when a rare condition occurred. Huang et al. used a finite state machine and majority vote whereby a state transition was only executed when a valid transition condition was met [87]. In terms of accuracy of control, majority vote does seem to provide accurate enough results, but it can add unacceptable delays to the system, which makes it hard to use in real time. Finding and implementing more post processing methods will be an inevitable part of advancing this field.

##### Performance metrics:

Deriving performance metrics for classification systems is an important step to evaluate the performance of the control system. There are different ways to evaluate a classification algorithm in upper limb control such as the TAC test [93] and Motion Test [80], [94]. These are not frequently reported in lower limb control due to the aforementioned quasi-cyclic motion of the limb. Instead, reported metrics for lower limb control were accuracy/error calculated as a ratio of correctly classified testing samples divided by the total number of applied testing samples. The extent to which offline performance measures represent real-time control performance is questionable for upper-limb control approaches [80], [95]. This is also an open question when quantifying performance in lower-limb control systems. An added complication is that the user needs the device to function properly to ambulate; the device needs to hold the person up while they ambulate. If the device does not function properly, the person may not be able to even attempt the activity, or transition between activities. An important metric was the number of missed locomotion transitions or critical error (an error that causes the user to feel unstable) [96]. While few studies have investigated the performance between offline and online metrics, it appears that they are correlated [46]. More work needs to be completed in this area and a platform in which to compare control methods using the universal metrics would be an advantage.

## IV. Challenges

The following summary outlines reported challenges that contemporary lower limb technology has not yet overcome.

### EMG signal quality and data acquisition:

EMG signal quality relies on factors such as electrode interface, availability of muscles, and the acquisition system [97]. Gradual variation of EMG signals over time is another challenge resulting from physical (electrode shift and impedance change) and/or physiological (human adaption and muscle fatigue) changes resulting in signal quality decay [54]. An implant surgically connected directly to the skeleton (via osseointegration) onto which an artificial limb can be attached is an alternative to a prosthetic socket. This connection method has been successfully used as a gateway to collect iEMG signals from research participants [18]. As a result, the deleterious effects of the prosthetic socket on sEMG is avoided and a much higher quality and more robust EMG signal can be obtained [22].

### Terrain transition detection:

One of the most challenging parts of controlling a prosthesis is transition detection, as it should be accurate and in a timely manner to control the prosthesis safely and smoothly [52], [69], [87]. There is potential to improve the accuracy of transition detection; perhaps by employing some of the techniques described in this review such as the conditional post processing technique used by Huang et al. [87]. Promising advancements in accuracy are expected with the use of iEMG. It may be that as the field moves towards this paradigm for EMG signal production (whether through an osseointegrated implant or via the implantation of wireless iEMG) that an improvement in terrain transition detection is obtained [18], [98].

### Stumbling:

It is critical to have a fast stumble detection method in lower limb control to avoid falling and subsequent injury [99]. Another challenge is handling perturbations during normal gait, such as slipping on a wet surface [100], which presents a challenge maintaining balance with a prosthetic leg. Transition and stumble detection and prevention can be improved by acquiring high quality EMG signal in combination with mechanical sensor signals. In addition to improved hardware, and more optimized software, it would help the development of the field if there were a dedicated platform on which to compare control methods in a scientific manner. Developing in this way by building on the work of previous groups would accelerate the field in our understanding of how to improve accuracy and reliability of these control algorithms.

## V. Conclusion

We reviewed the literature on the control of powered prosthetic legs using EMG. We described work undertaken in direct control, model-based, and machine learning methods. Of the reviewed literature direct control and machine learning methods for control have produced favorable results in transtibial and transfemoral research participants. However, it remains challenging to control a prosthetic leg with EMG signal in an environment outside the lab, such as at the home (the ultimate goal) and particularly for transfemoral amputations. Direct control is frequently reported but is only suitable for a few degrees of freedom; more complex movement intentions can be decoded using machine learning methods. Looking forwards, we think machine learning methods should be the focus for the control of lower limb prostheses with EMG signals.

In addition to control methods, we described the activity types, electrode placement, and how EMG based control was augmented with non-biological sensors. Weight bearing tasks were predominantly reported in the literature, but there was large variability in the number of research participants and electrode positioning. Foot switches and load cells were the most often reported mechanical sensors that were used in combination with EMG signals.

We paid special consideration to studies using real-time decoding of locomotion modes since this better reflects homeuse of a prosthesis. The goal of this review was to present available technologies and to highlight the opportunities in the field of lower limb prosthetic control using myoelectric signals. However, there was a challenge comparing studies due to the large variability in methods and outcome metrics; in future work a standardization of these would be useful.

## Figures and Tables

**TABLE I:** Summary of Research Studies Using Direct Control

TITLE	RESEARCH SUBJECTS	SUBJECT ACTIVITY	CONTROL SOURCE	REAL-TIME/OFFLINE	TRANSITION DETECTION	#EMG Channels
[70]	AB/TF	Weight bearing	EMG + load cell, joint motion sensor, force sensitive resistors	Real-time/offline	No	8
[101]	TF	Weight bearing	EMG +load cell, force sensitive resistors	Real-time	No	8
[102]	AB/TF	Weight bearing	EMG	Real-time	No	2
[103]	AB	Weight bearing	EMG + force resistors	Real-time	No	5
[104]	TT	Weight bearing	EMG	Real-time	No	7
[105]	TT	Weight bearing	EMG + IMU, loadcell	Real-time/offline	Yes	2
[106]	AB	Weight bearing	EMG +force resistors	Real-time	No	5
[61]	AB/TF	Weight bearing	EMG + knee angle sensor, pressure sensor, torque sensor,	Real-time	No	1
[107]	AB	Non- weight bearing	EMG	Real-time	No	8
[71]	AB	Non- weight bearing	EMG	Real-time	No	2
[108]	TT	Weight bearing	EMG	Real-time	No	2
[40]	AB/TT	Non- weight bearing	EMG	Real-time	No	NI
[33]	AB/TT	Non- weight bearing	EMG	Real-time	No	2
[109]	TT	Weight bearing	EMG	Real-time	No	NI
[43]	TT	Weight bearing	EMG	Real-time	No	2
[11]	TT	Non-weight bearing	EMG	Real-time	No	4
[36]	AB	Non-weight bearing	EMG	Real-time	No	2
[69]	TT	Weight bearing	EMG	Real-time	Yes	2
[41]	TT	Non-weight bearing	EMG	Real-time	No	NI
[110]	TT	Weight bearing	EMG	Offline	No	NI
[111]	TF	Weight bearing	EMG	Real-time	No	NI
[112]	TT	Weight bearing	EMG	Real-time	No	NI
[113]	TT	Weight bearing	EMG	Real-time	No	192
[114]	TT	Weight bearing	EMG	Real-time	No	>2

TF: Research participants with transfemoral amputation, TT: Research participants with transtibial amputation, Ab: Able-bodied research participants

**TABLE II:** Summary of Research Studies Using Phase Dependent Classifier

Title	Amputation Level	Activity	Control /Classification algorithm	Transition Detection(Y/N)	Real-Time/Offline	# EMG Channels
[28]	TF/AB	Level-ground walking, ramps, and stairs	Entropy-based adaptation (EBA), Support Vector Machine (SVM)	Y	Real-Time/Offline	16
[29]	TF	Level-ground walking, ramps, and stairs	Baseline Configuration, Time History Configuration, Mode-Specific Configuration, Mode Specific and Time History Configuration	Y	Offline	NI
[88]	TF	Level-ground walking, ramps, and stairs	Linear Discriminant Analysis (LDA)	Y	Real-Time/Offline	10
[44]	TF	Level-ground walking, ramps, and stairs	Dynamic Bayesian Network (DBN), LDA	Y	Offline	9
[45]	TF	Level-ground walking, ramps, and stairs	LDA and log likelihood threshold	Y	Offline	8
[86]	TF	Level-ground walking, ramps, and stairs, stepping over an obstacle	LDA	Y	Offline	16
[46]	TF	Level-ground walking, ramps, and stairs	LDA and DBN	Y	Real-Time/Offline	9
[30]	TF	Level-ground walking, ramps, and stairs	Forward- Backward state machine	Y	Real-Time	8
[47]	TF	Sitting, standing, walking	LDA	Y	Real-Time/Offline	8
[83]	TF	Level-ground walking, ramps, and stairs sitting, standing	SVM	Y	Real-Time	10
[10]	TF	Level-ground walking, ramps, and stairs	Dynamic Bayesian Network	Y	Real-Time	96
[48]	TF	Level-ground walking, stair, ramp, sitting, and standing.	SVM, C-support vector classification (C-SVC)	Y	Real-Time/Offline	10
[64]	TT/TF	Level-ground walking, ramps, and stepping over an obstacle	Bayes Classifier	N	Offline	11
[115]	AB	Walking	Back propagation neural network	N	Offline	8
[49]	TF/AB	Level-ground walking, stepping over an obstacle, stairs, turning, and standing still	LDA	N	Offline	16
[67]	TF	Level-ground, ramps, and stairs	LDA	Y	Offline	9
[50]	TF	Level-ground walking, ramps, and stairs	DBN	Y	Offline	9
[116]	AB	Level-ground walking, ramps, and stairs	Hidden Markov Model (HMM)	Y	Offline	4
[117]	TF	Level-ground walking, ramps, and stairs	LDA	Y	Offline	9
[51]	TF	Level-ground walking, ramps, and stairs, stepping over an obstacle	SVM	Y	Real-Time	16
[52]	TF	Level ground, ramps, and stairs	DBN	Y	Offline	9
[63]	TF	Level-ground walking, ramps, and stairs	LDA	Y	Offline	1
[54]	TF	Level-ground walking, ramps, and stairs	SVM	Y	Offline	7
[87]	AB	Sitting and standing	LDA	N	Real-Time/Offline	7
[89]	AB	level-ground walking, ramps, and stairs	LDA	Y	Offline	5
[118]	AB	Level walking	SVM	N	Offline	4
[58]	TT/AB	Level ground at three speeds, ramp ascent/descent, and stair ascenl/descent.	LDA, SVM	N	Offline	4
[55]	TF	Walking, stairs sitting and standing	Binary tree	Y	Offline	6
[119]	AB	Level-ground walking, ramps, and stairs	Unscented Kalman Filter	N	Offline	6
[83]	TF	Level-ground walking, ramps, and stairs	SVM	Y	Real-Time/Offline	10
[120]	AB	Walking with different speeds	Neural network, Genetic Algorithms	N	Offline	5
[77]	AB	Standing and walking on slope, flat and stair	Neural network	N	Offline	NI
[59]	AB	Walking	LDA	N	offline	14
[85]	AB	Level-ground walking, ramps, and stairs	LDA, SVM	Y	Offline	15
[121]	TF/AB	Walking	LDA	N	Offline	6
[99]	TF	Walking with obstacle	Mahalanobis distance	N	Offline	10
[92]	AB	Level-ground walking, ramps, and stairs, standing	SVM	Y	Real-Time/Offline	NI
[122]	TT	Walking	Neural network with exogenous input (NARX)	Y	Offline	5
[53]	TF	Walking, stairs, ramps	SVM, LDA, ANN	Y	Offline	14
[62]	AB	Walking, stairs, ramps	LDA, SVM, NN	Y	Offline	12
[57]	TT	Dorsiflexion and plantarflexion	LDA	N	Offline	5

TF: Research participants with transfemoral amputation, TT: Research participants with transtibial amputation, Ab: Able-bodied subjects

**TABLE III:** Summary of Research Studies Using Continuous Classifier

Title	Amputation Level	Activity	Control /Classification algorithm	Transition Detection(Y/N)	Real-Time/Offline	# EMG Channels
[123]	AB	Plantar-flexion and dorsiflexion	Support Vector Machine (SVM)	N	Offline	9
[124]	AB	Walking, ascending Stairs	Matching scores (MS) and discriminating functions in the form of simple if-else rules	Y	Offline	NI
[125]	AB	Walking, stair Ascend, stair Descend, ramp ascend, and ramp descend	SVM, Linear Discriminant Analysis (LDA), and Neural network (NN)	N	Offline	NI
[32]	TT	Mimic pre-programmed motion trajectories of the graphical display	NN	N	Offline	2
[57]	TT	Dorsiflexion and plantarflexion movements with a resting pause in-between	LDA	N	Offline	5
[126]	AB	Level Walking, Stair Ascent, Stair Descent, Ramp Ascent, and Ramp Descent	NN	N	offline	5
[127]	AB	Walking	NN	N	offline	2
[128]	AB	Walking, Stair Ascend, and Stair Descend	SVM, LDA, ANN, decision tree (DT), k-nearest neighbor (KNN) and naive bias (NB) and k-fold cross validation		Offline	NI
[78]	AB	Sitting, standing up	Packet wavelet	N	Offline	4
[84]	AB	Walking, stairs	LDA, DT, KNN, NB, NN, SVM	N	Offline	NI
[66]	AB	Walking	LDA	N	Offline	4
[129]	TF	Walking	NN	N	Offline	2
[130]	AB	Walking	NN	-	Offline	4
[131]	AB	Walking	NN	N	Offline	2
[91]	AB	Walked at three self-selected speeds, level-ground and a custom made fixed, uneven terrain shown	LDA	N	Offline	8
[132]	AB	Walking	NN	N	Offline	2
[133]	AB	Walk with different pace	LDA, ANN, NB	N	Offline	3
[134]	AB	Classification between rest and beginning of movement	Hilbert Huang transfer	N	Offline	NI
[90]	AB	Walking	LDA, SVM	N	Offline	4
[38]	TF/AB	Knee flexion, knee extension, ankle plantar flexion, ankle dorsiflexion, femoral rotation, and tibial rotation	LDA	N	Real-Time/Offline	9
[135]	TF/AB	Sitting, standing	LDA, Dynamic Pattern Classification Strategy	N	Real-Time	7
[136]	AB	Walking with three different speeds	NN, SVM	N	Offline	3
[137]	AB	Upstairs, downstairs, uphill and downhill	Wavelet	N	Offline	2
[138]	AB	Standing up from a chair and sitting down on the chair	Naive Bayes, k-nearest neighbor, SVM	N	Offline	4
[139]	AB	Walking modes with three different speeds	Neural network	N	Offline	5
[140]	AB	Walking, stairs	DT, KNN, NN, SVM, LDA	N	Offline	2
[141]	AB	Cycling	NN	N	Offline	7
[142]	AB	Walking, Stairs	LDA, SVM, NN	Y	offline	5
[143]	AB	Standing, Walking, Running	ANN, dynamic recurrent neural network (DRNN)	N	Offline	6
[37]	AB	Knee extension/flexion, femur rotation in/ out, tibia rotation in/out and ankle dorsiflexion/plantarflexion	LDA	N	offline	8
[101]	TF	Standing, squatting, and sit-stand transitions	Impedance control	N	Real-Time	8
[56]	TF/AB	Rotation of the foot, ankle dorsiflexion and ankle plantarflexion, ankle eversion and ankle inversion (or ankle pronation/supination)	LDA	-	Real-Time/Offline	NI
[144]	TF/TT/AB	Walking, ramps	SVM, LDA		Offline	5
[145]	AB	Ankle and knee movements	CNN (Convolutional Neural Network)	-	Real-Time	4
[146]	AB	Walking, stairs, ramps	NN	N	Offline	4

TF: Research participants with transfemoral amputation, TT: Research participants with transtibial amputation, Ab: Able-bodied subjects
